# Risk of secondary stroke subsequent to restarting aspirin in chronic stroke patients suffering from traumatic brain injury in Taiwan

**DOI:** 10.1038/s41598-023-34986-z

**Published:** 2023-05-17

**Authors:** Chu-Lin Chou, Chi-Hsiang Chung, Yung-Ho Hsu, Chia-Chao Wu, Chien-An Sun, Wu-Chien Chien, Shih-En Tang, Li-Yun Fann

**Affiliations:** 1 Division of Nephrology, Department of Internal Medicine, Tri-Service General Hospital, National Defense Medical Center, Taipei, Taiwan, Republic of China; 2 Division of Nephrology, Department of Internal Medicine, School of Medicine, College of Medicine, Taipei Medical University, Taipei, Taiwan, Republic of China; 3 Taipei Medical University-Research Center of Urology and Kidney, Taipei Medical University, Taipei, Taiwan, Republic of China; 4 Division of Nephrology, Department of Internal Medicine, Hsin Kuo Min Hospital, Taipei Medical University, Taoyuan City, Taiwan, Republic of China; 5 Division of Nephrology, Department of Internal Medicine, Shuang Ho Hospital, Taipei Medical University, New Taipei City, Taiwan, Republic of China; 6 School of Public Health, National Defense Medical Center, Taipei, Taiwan, Republic of China; 7 Taiwanese Injury Prevention and Safety Promotion Association, Taipei, Taiwan, Republic of China; 8 Department of Public Health, College of Medicine, Fu-Jen Catholic University, New Taipei City, Taiwan, Republic of China; 9 Big Data Research Center, College of Medicine, Fu-Jen Catholic University, New Taipei City, Taiwan, Republic of China; 10 Department of Medical Research, Tri-Service General Hospital, National Defense Medical Center, 7115R, No. 325, Section 2, Cheng-Kung Road, Neihu District, Taipei City, 11490 Taiwan, Republic of China; 11 Graduate Institute of Life Sciences, National Defense Medical Center, Taipei, Taiwan, Republic of China; 12 Department of Internal Medicine, Division of Pulmonary and Critical Care Medicine, Tri-Service General Hospital, National Defense Medical Center, Taipei, Taiwan, Republic of China; 13 Institute of Aerospace and Undersea Medicine, National Defense Medical Center, Taipei, Taiwan, Republic of China; 14 Department of Nursing, Taipei City Hospital, Taipei, Taiwan, Republic of China; 15 Department of Nurse-Midwifery and Women Health, National Taipei University of Nursing and Health Sciences, Taipei, Taiwan, Republic of China

**Keywords:** Brain injuries, Cerebrovascular disorders, Stroke, Drug safety

## Abstract

Traumatic brain injury (TBI) is a silent epidemic that has been easily ignored. The safety and efficacy of restarting antiplatelet therapy after encountering traumatic brain injury (TBI) events remain a challenge. We explored the outcomes of restarting aspirin use on secondary stroke and mortality in patients with chronic stroke 4 weeks after suffering from a TBI episode in Taiwan. This study analyzed data from the National Health Insurance Research Database from January 2000 to December 2015. Overall, 136,211 individuals diagnosed with chronic stroke who suffered from acute TBI and received inpatient service were enrolled. The study outcomes were a competing risk of secondary stroke (ischemic and hemorrhagic) hospitalization and all-cause mortality. We identified a case group of 15,035 patients with chronic stroke (mean [SD] age of 53.25 [19.74] years; 55.63% male) who restarted aspirin use 4 weeks after suffering from TBI and a control group of 60,140 patients with chronic stroke (mean [SD] age of 53.12 [19.22] years; 55.63% male) who discontinued aspirin use after suffering from TBI. The risk of hospitalization of secondary ischemic stroke [adjusted hazard ratio (aHR) 0.694; 95% confidence interval (CI) 0.621–0.756; *P* < 0.001] and hemorrhagic stroke (aHR 0.642; 95% CI 0.549–0.723; *P* < 0.001) and all-cause mortality (aHR 0.840; 95% CI 0.720–0.946; *P* < 0.001) significantly decreased in patients with chronic stroke restarting aspirin use 1 month after suffering from TBI events (including intracranial hemorrhage) in comparison with the control subjects, regardless of those with or without diabetes mellitus, chronic kidney disease, myocardial infarction, atrial fibrillation, clopidogrel use, and dipyridamole use. Restarting aspirin use could lower the risks of secondary stroke (ischemic and hemorrhagic) hospitalization and all-cause mortality in patients with chronic stroke 1 month after suffering from TBI episodes.

## Introduction

Acute stroke, the most severe and disabling neurological disorder worldwide, is a crucial public health concern, and over the past decades, there have been progress in prevention and treatment. The benefits of aspirin in preventing early recurrent ischemic changes are more significant than earlier recognized^1^. Other strategies to prevent acute stroke comprise alternative anticoagulants warfarin for atrial fibrillation, intravenous alteplase, and carotid stenting with or without endovascular thrombectomy for treating acute ischemic stage^2^. Pharmacological and interventional therapies promise to facilitate brain rehabilitation and functional recovery^3,4^. Despite decreasing stroke mortality through these treatments, the global burden of acute stroke is on the rise. A comprehensive approach targeting people at risk for other diseases who share common risk factors to prevent secondary stroke is obligatory.

Traumatic brain injury (TBI) is a silent epidemic that has been easily ignored^5^; it is associated with epilepsy^6^, cognitive decline^7,8^, and psychiatric conditions^9^. TBI has also been reported to be associated with a tenfold risk of stroke within 3 months, such as risks of increasing ischemic stroke and intracerebral hemorrhage, according to a study with a total of 23,199 patients^10^. It has been postulated that a TBI causes brain damage, further involving the loss of brain functions by triggering disturbances in the blood supply to the brain and leading to a stroke^11^. Patients with TBI often experience traumatic cerebral microbleeds (also known as hemorrhagic diffuse axonal injury) associated with the severity of the injury, especially in moderate-to-severe traumatic brain injury patients. Brain microbeads are useful markers for determining the nature and severity of the underlying chronic condition of small vessels^12^. Moreover, Microbleeds International Collaborative Network, new risk scores incorporating clinical variables and cerebral microbleeds, provide predictive value with the long-term risks of intracranial hemorrhage and ischaemic stroke in patients with chronic stroke for secondary stroke prevention^13^. Thus, we need initiatives to raise patient and public awareness about the risk of stroke following a TBI, especially in the first few months.

However, up to date, there is little evidence of the outcome of restarting aspirin use on secondary stroke in patients with chronic stroke following a TBI episode. In this study, using the National Health Insurance Research Database (NHIRD) in Taiwan, we explored the outcomes of secondary stroke (ischemic and hemorrhagic) and all-cause mortality in patients with chronic stroke with and without starting aspirin use 4 weeks after suffering from a TBI episode in Taiwan.

## Methods

### Data source

The Taiwan National Health Insurance system is a universal single-payer insurance system and enrolled all these insurance schemes into a single national insurance system. The NHIRD provides longitudinal databases which randomly sampled two million beneficiaries from the original NHIRD^14^. The representativeness of NHIRD has been validated by Taiwan’s National Health Research Institutes^14^. Informed consent is waived due to personal information that had been de-identified in the NHIRD and informed consent waiver was approved by the Institutional Review Board of the Tri-Service General Hospital (IRB Number: B-109-36). All information allowing an enrolled patient to be identified was encrypted. This study was carried out in accordance with the approved protocol and the Declaration of Helsinki. All experimental protocols were approved by the Institutional Review Board of the Tri-Service General Hospital, Taipei, Taiwan. A request for the analytic methods should be sent to the corresponding author.

This retrospective study investigated all patients diagnosed with chronic stroke who suffered from a TBI episode and received inpatient service linked with the NHIRD. The time interval between chronic stroke and TBI was within half one year. All patients were identified based on the International Classification of Diseases, Ninth Revision, Clinical Modification (ICD-9-CM) codes in the Registry of Catastrophic Illness in 2000. Information on the study's identification was collected within 15 years—specifically, data were recorded from January 2000 to December 2015.

### Study design and outcomes

Using the Taiwan NHIRD, patients with chronic stroke following a TBI episode and who received inpatient service were collected in the following two groups: aspirin users and nonusers. The definition of TBI included intracranial hemorrhage at the same time (Supplemental Table S1). This study was conducted using the inpatient and outpatient claims data recorded from January 2000 to December 2015. The aspirin group was made up of patients with chronic stroke in whom aspirin use was restarted 4 weeks after suffering from a TBI episode. The control group was made up of patients with chronic stroke in whom aspirin use was still discontinued after they suffered from a TBI episode.

We excluded patients whose ages and genders were not recorded, who were younger than 40 years, who had a minimum of 2 years of unavailable data following TBI initiation, who were chronic stroke and in whom aspirin use was not prescribed within 6 months before suffering from TBI, who had a history of cancer and human immunodeficiency virus, and anticoagulation (warfarin and rivaroxaban) user.

The index date was defined as the start of aspirin use at 4 weeks after suffering from a TBI episode in patients with chronic stroke. The study follow-up period started from the index date to the onset date of secondary stroke (ischemic and hemorrhagic) hospitalization and all-cause mortality. We assigned a date for the control patients who did not continue aspirin use in chronic stroke after suffering from a TBI episode, which matched their corresponding case patients (referred to as the index date). Because controls did not have aspirin use, they were assigned a date for a pseudo-aspirin event, which corresponded to the index date of their matched patients (referred to as the index date hereafter).

Finally, cases and controls were all matched for age, gender, and covariates, including comorbidities and medications that had continued use for 6 months before suffering from a TBI episode (Supplemental Table S1). We used the incidence density sampling approach to match controls with each case according to age (± 1 year), sex, and the follow-up period of aspirin use in patients with chronic stroke after suffering from TBI. This approach allowed us to observe both patient groups for similar periods, thus eliminating the bias caused by time frame differences.

The study outcomes were the competing risk of secondary stroke (ischemic and hemorrhagic) hospitalization and all-cause mortality. The outcome of interest was the first hospitalization for stroke causes from the NHI claims database after study initiation. All-cause mortality within the 15 years follow-up period was considered an event of interest. The causes of mortality after study initiation, such as ischemia stroke, hemorrhagic stroke, myocardial infarction, arrhythmia, out-of-hospital cardiac arrest, and infectious disease, were also assessed according to the primary diagnostic codes entered in the inpatient claims and emergency claims 30 days before death. Patients were followed up from the index date to the earliest of the following: outcome occurrence, death, disenrollment of the NHI program, or December 31, 2015.

### Covariates

Baseline demographic and clinical characteristics recorded before the index date were obtained. The comorbidities included diabetes mellitus (DM), chronic kidney disease (CKD), myocardial infarction, atrial fibrillation, hypertension, hyperlipidemia, heart failure, peripheral vascular disease, chronic pulmonary disease, chronic liver disease, and dementia. We measured the risk of outcomes in patients who had received medications within 6 months before the index date. The prescribed medications included DM and non-DM medications. The major classes of DM medications included metformin, sulfonylureas, thiazolidinediones, a-glucosidase inhibitor, dipeptidyl peptidase 4 inhibitors, and insulin. The non-DM medications included clopidogrel, dipyridamole, antiplatelet drug, angiotensin-converting enzyme inhibitors/angiotensin receptor blockers, beta-blockers, calcium channel blockers, statins, and nonsteroidal anti-inflammatory drugs. The ICD-9-CM disease diagnostic codes used for previous or coexisting diseases and the Anatomical Therapeutic Chemical codes used for medications are listed in Supplemental Table S1.

### Statistical analysis

We applied propensity score matching to balance the two groups with respect to known confounders and to ensure comparability during analysis^15^. Propensity score methods for reducing the effects of confounding in observational studies mimic some of the particular characteristics of a randomized controlled trial^16^. We adjusted for potential confounding variables of the comorbidities, DM medications, and non-DM medication on the basis of clinical relevance. Continuous variables are reported as mean (SD) depending on the distribution of the data. Categorical variables are reported as numbers and percentages. The patients’ baseline characteristics were compared using standardized differences that reflect the mean difference as a percentage of the standard deviation, as indicated by Mamdani et al.^17^. We compared baseline characteristics between the group that restarted aspirin for chronic stroke patients who suffered from TBI episodes in the matched control group using standardized differences, with a standardized difference greater than 10% considered meaningful. All variables in the analysis were complete, which were missing for less than 0.5% of the cohort. The SAS statistical software (SAS System for Windows, version 9.1.3; SAS Institute, Cary, NC, USA) was used for statistical analyses. The χ^2^ test and *t*-test were used to analyze and evaluate the differences in age or comorbidities and medications between the aspirin and non-aspirin groups. The Fisher's exact test was applied for categorical variables to statistically compare the differences between the two cohorts. The Cox proportional regression hazards model was used to compare the incidence rates of secondary stroke (ischemic and hemorrhagic) hospitalization and all-cause mortality between the aspirin and non-aspirin groups after the modification of comorbidities. We tested the proportionality assumption by adding a time-dependent exposure covariate to the model. The Kaplan–Meier method and log-rank test were used to estimate the outcomes of the two groups. A two-tailed *P* value of < 0.05 was considered statistically significant.

## Results

### Baseline characteristics

**Figure 1:**
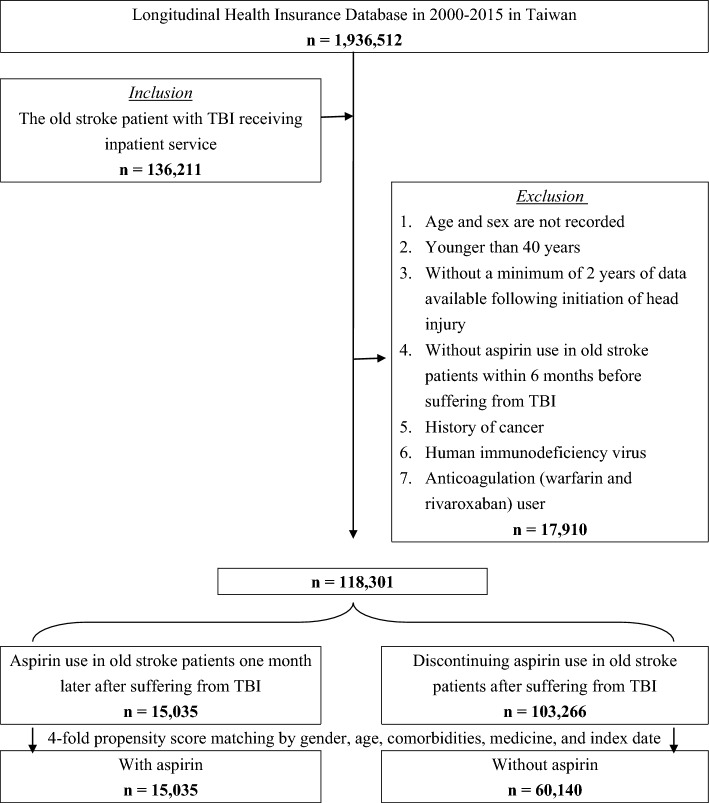
Case assembly in this study. *TBI* traumatic brain injury.

A total of 136,211 individuals diagnosed with chronic stroke who suffered from TBI for the first time and received inpatient service were enrolled in this study, as shown in Fig. 1. This study had been conducted from January 2000 to December 2015. Of these individuals, 17,910 patients with chronic stroke suffering from TBI were excluded [including patients whose ages and genders were not recorded, those younger than 40 years, without at least 2 years of data available following TBI initiation, without aspirin use in chronic stroke patients within 6 months before suffering from head TBI, a history of cancer and human immunodeficiency virus, and anticoagulation (warfarin and rivaroxaban) user]. The baseline characteristics of those subjects before using the propensity score matching was showed in Supplemental Table S2. After using the propensity score matching according to the index date, gender, age, comorbidities, and medications, we identified a case group of 15,035 patients with chronic stroke (mean [SD] age of 53.25 [19.74] years; 55.63% male) who restarted aspirin use 1 month after suffering from TBI and a control group of 60,140 patients with chronic stroke (mean [SD] age of 53.12 [19.22] years; 55.63% male) who discontinued aspirin use after suffering from TBI. Table 1 shows the baseline characteristics of patients with chronic stroke who did or did not receive aspirin following a TBI episode after using the propensity score matching. There were no significant differences in gender, age, and all covariates, including comorbidities and medications between the groups. The standardized differences before and after using the propensity score matching was showed in Supplemental Table S3. In this study, the mean ± standard deviation of follow-up was 9.24 ± 5.17 years, with a minimum of 0.01 years and a maximum of 15.87 years, and the median was 7.22 years.

**Table 1:** Baseline characteristics of study subjects.

Aspirin	With (n = 15,035)	Without (n = 60,140)	*P*
Characteristics	Mean ± SD	Mean ± SD	
Age (years)	53.25 ± 19.74	53.12 ± 19.22	0.412
	No. (%)	No. (%)	
Sex			0.999
Male	8364 (55.63)	33,486 (55.63)	
Female	6,671 (44.37)	26,684 (44.37)	
Comorbodities			
Diabetes mellitus	3784 (25.17)	15,382 (25.58)	0.271
CKD	737 (4.90)	2859 (4.75)	0.403
Myocardial infarction	3509 (23.34)	13,977 (23.24)	0.768
Atrial fibrillation	1040 (6.92)	4192 (6.97)	0.835
Hypertension	4838 (32.18)	19,320 (32.13)	0.926
Hyperlipidemia	1146 (7.62)	4528 (7.53)	0.281
Heart failure	198 (1.32)	881 (1.46)	0.173
Peripheral vascular disease	899 (5.98)	3614 (6.01)	0.909
Chronic pulmonary disease	1615 (10.74)	6429 (10.69)	0.852
Chronic liver disease	2490 (16.56)	9683 (16.10)	0.703
Dementia	625 (4.16)	2406 (4.00)	0.411
Medication			
Clopidogrel	2225 (14.80)	8774 (14.59)	0.477
Dipyridamole	2458 (16.35)	9520 (15.83)	0.093
ACEI/ARB	4370 (29.07)	17,542 (29.17)	0.862
Beta-2 blocker	3755 (24.98)	14,771 (24.56)	0.283
CCB	2646 (17.60)	14,574 (24.23)	0.072
Antiplatelet drug	4552 (30.28)	18,312 (30.45)	0.796
Statin	4371 (29.07)	17,662 (29.37)	0.548
NSAID	3024 (17.60)	10,765 (17.90)	0.382
Metformin	4511 (30.00)	18,142 (30.17)	0.761
Thiazolidinedione	4106 (16.24)	17,169 (28.55)	0.185
Sulfonylureas	3628 (24.13)	14,643 (24.35)	0.498
Alpha-glucosidase inhibitor	2441 (16.24)	9758 (16.23)	0.983
DPP4is	3255 (21.65)	12,973 (21.57)	0.788
Insulin	2357 (15.68)	9363 (15.57)	0.72

*P*: Chi-square/Fisher exact test on category variables and t-test on continue variables.

### Effects of restarting aspirin use on secondary stroke 4 weeks after suffering from TBI episodes in chronic stroke patients

**Figure 2:**
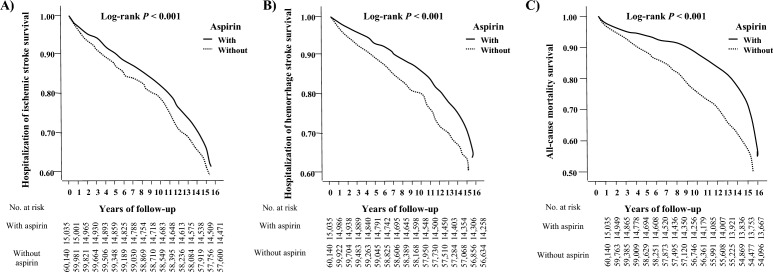
Kaplan–Meier curves for the cumulative incidence of secondary stroke [(**A**) ischemic stroke and (**B**) hemorrhagic stroke] hospitalization and (**C**) all-cause mortality in chronic stroke patients with traumatic brain injury with aspirin use or without aspirin use.

The cumulative incidence of secondary stroke (ischemic and hemorrhagic) hospitalization and all-cause mortality in the Cox model with competing risks are shown in Table 2 and Fig. 2. The competing risk of hospitalization of secondary ischemic stroke [adjusted hazard ratio (aHR), 0.694; 95% confidence interval (CI) 0.621–0.756; *P* < 0.001] and hemorrhagic stroke (aHR 0.642; 95% CI 0.549–0.723; *P* < 0.001) and all-cause mortality (aHR 0.840; 95% CI 0.720–0.946; *P* < 0.001) in patients with chronic stroke restarting aspirin use 4 weeks after suffering from TBI episodes significantly decreased in comparison with the control subjects during the follow-up period after adjusting for gender, age, comorbidities, and medications in Taiwan.

**Table 2:** Hazard ratio of hospitalization (ischemic stroke and hemorrhagic stroke) and mortality in association with baseline characteristics in chronic stroke patients with traumatic brain injury with aspirin use or without aspirin use in the Cox model with competing risks.

Fine and Gray competing risk	With aspirin		Without aspirin		With aspirin versus without aspirin (Reference)							
					No competing in the model				Competing risk in the model			
Outcomes	Events	PYs	Events	PYs	aHR	95% CI	95% CI	*P*	aSHR	95% CI	95% CI	*P*
Hospitalization of stroke	1340	9.59	6,046	10.89	0.671	0.591	0.731	< 0.001	0.679	0.606	0.742	< 0.001
Hospitalization of ischemic stroke	697	4.99	3,134	5.64	0.679	0.612	0.745	< 0.001	0.694	0.621	0.756	< 0.001
Hospitalization of hemorrhage stroke	643	4.60	2,912	5.24	0.631	0.538	0.720	< 0.001	0.642	0.549	0.723	< 0.001
All-caused mortality	1564	9.13	5,956	10.06	0.840	0.720	0.946	< 0.001	–	–	–	–

Rate: per 1000 Rate: per 1000 PYs; aHR = Adjusted Hazard ratio: Adjusted for gender, age, Comorbodities, and medications; CI = confidence interval; aSHR = Adjusted Subdistribution Hazard Ratio: Adjusted for gender, age, Comorbodities, and medications.

Furthermore, we performed a subgroup analysis of the association between aspirin use and risks of secondary stroke (ischemic and hemorrhagic) hospitalization and all-cause mortality (Supplemental Tables S4 and S5). Regardless of their underlying diseases, including DM, CKD, myocardial infarction, and atrial fibrillation, and medications, including clopidogrel and dipyridamole, restarting aspirin use still had beneficial effects on lowering the risks of secondary stroke hospitalization (ischemia and hemorrhage) in patients with chronic stroke 1 month after TBI, as compared with that in the matched control group that discontinued aspirin use after TBI (Supplemental Table S4). For all-cause mortality, moreover, restarting aspirin was associated with decreased death risk in patients with and without DM, myocardial infarction, and medications, including clopidogrel and dipyridamole use (Supplemental Table S5).

Cumulative dosages of aspirin were also associated with reducing the risk of hospitalization (ischemic and hemorrhagic stroke) and all-cause mortality in those patients with TBI episodes, as shown in Table 3, respectively. Ischemic stroke, hemorrhagic stroke, and myocardial infarction were significantly associated with the causes of all-cause mortality after restarting aspirin use in patients with chronic stroke 1 month after TBI episodes (Supplemental Table S6).

**Table 3:** Cumulative defined daily dose (cDDD) exposure of aspirin and the risk of hospitalization (ischemic and hemorrhagic stroke) and all-cause mortality.

Fine and gray competing risk		Events	Rate	No competing in the model				Competing risk in the model			
Outcomes	cDDD of aspirin			aHR	95% CI	95% CI	*P*	aSHR	95% CI	95% CI	*P*
Hospitalization of stroke	Without	6046	10.89	*Reference*				*Reference*			
	< 90	481	10.33	0.814	0.681	0.926	< 0.001	0.823	0.690	0.963	0.018
	90–364	436	9.36	0.735	0.644	0.794	< 0.001	0.746	0.661	0.817	< 0.001
	≧365	423	9.08	0.581	0.450	0.691	< 0.001	0.603	0.487	0.712	< 0.001
Hospitalization of ischemic stroke	Without	3134	5.64	*Reference*				*Reference*			
	< 90	243	5.22	0.829	0.702	1.050	0.082	0.842	0.707	1.076	0.107
	90–364	231	4.96	0.737	0.666	0.822	< 0.001	0.762	0.666	0.830	< 0.001
	≧365	223	4.79	0.592	0.472	0.707	< 0.001	0.612	0.515	0.718	< 0.001
Hospitalization of hemorrhage stroke	Without	2912	5.24	*Reference*				*Reference*			
	< 90	238	5.11	0.740	0.611	0.825	< 0.001	0.742	0.567	0.860	< 0.001
	90–364	205	4.40	0.687	0.585	0.788	< 0.001	0.693	0.544	0.796	< 0.001
	≧365	200	4.29	0.556	0.397	0.614	< 0.001	0.566	0.407	0.692	< 0.001
All-caused mortality	Without	5956	10.06	*Reference*				–	–	–	–
	< 90	531	10.78	1.040	–	–	–	–	–	–	–
	90–364	420	8.52	0.940	–	–	–	–	–	–	–
	≧365	399	8.10	0.764	–	–	–	–	–	–	–

Rate: per 1000 Rate: per 1000 PYs; aHR = Adjusted Hazard ratio: Adjusted for gender, age, Comorbodities, and medications; CI = confidence interval; aSHR = Adjusted Subdistribution Hazard Ratio: Adjusted for gender, age, Comorbodities, and medications.

Furthermore, we defined severe TBI as TBI cases with long-stay hospitalization or surgical treatment. As shown in Supplemental Table S7 for severe TBI, we found that besides all-cause mortality, there is a lower competing risk of hospitalization of secondary ischemic stroke and hemorrhagic stroke after restarting aspirin use 1 month after TBI episodes in patients with chronic stroke.

## Discussion

The purpose of aspirin use in patients with chronic stroke is to ameliorate stroke progression and secondary attack, but restarting aspirin use following TBI episodes remains a significant challenge, especially for hemorrhage tendency. Our study explored the outcomes of secondary stroke (ischemic and hemorrhagic) hospitalization and mortality in patients with chronic stroke with and without restarting aspirin use 4 weeks after suffering from a TBI episode in Taiwan. After multivariate adjustment and subgroup analysis, this study presented that restarting aspirin use is associated with reducing secondary stroke, including ischemic and hemorrhagic, and all-cause mortality in patients with chronic stroke with TBI episodes (including intracranial hemorrhage episodes) 4 weeks later. Regardless of those with or without DM, CKD, myocardial infarction, and atrial fibrillation and clopidogrel, and dipyridamole, restarting aspirin use remains safe and beneficial in lowering secondary stroke hospitalization and all-cause mortality in patients with chronic stroke 4 weeks after TBI episodes.

The safety and efficacy of restarting antiplatelet therapy after encountering TBI episodes remain a challenge. Despite expectations that restarting aspirin following episodes of hemorrhage-associated stroke might increase the risk for a recurrent brain hemorrhage, researchers have found the opposite^18–23^. For example, in the RESTART trial that recruited 573 subjects with chronic stroke following a brain hemorrhage, the researchers found that restarting antiplatelet agents did not increase the rate of a recurrent brain hemorrhage, which indicated that hemorrhagic episodes were found not to differ between the restart and avoid-antiplatelet therapy groups significantly^18^. Also, the RESTART study showed that restarting antiplatelet agents tended to reduce brain hemorrhage (adjusted HR, 0.51; 95% CI 0.25–1.03; *P* = 0.060); this effect seems to be primarily derived from the prevention of thrombotic events^19,21^. Similarly, our study observed that restarting aspirin therapy is associated with a low risk of hemorrhagic stroke in patients with chronic stroke suffering TBI episodes.

No expert consensus or strategies elucidated the benefits and risks of restarting aspirin in patients with chronic stroke with TBI episodes. Emerging report of a meta-analysis that combined observational cohort for 1916 patients showed that aspirin resumption had a lower risk for thromboembolic events (relative risk, 0.61; 95% CI 0.48–0.79; *P* < 0.01) without increasing the risk of brain hemorrhage recurrence in patients with primary intracranial hemorrhage^19^. Our study also indicated that restarting aspirin decreased the risk of ischemic stroke in patients with chronic stroke with TBI episodes, similar to those previously reported^19,24^. Furthermore, we observed that restarting aspirin benefited from survival in chronic stroke with TBI episodes, regardless of those with or without DM, myocardial infarction, and atrial fibrillation and clopidogrel, and dipyridamole. An unnecessary closing of aspirin therapy in those patients might increase the ischemic or thromboembolic risk. Thus, whether to restart aspirin uses or not requires physicians to balance ischemic and hemorrhagic risk.

The optimal timing of restarting antiplatelet therapy in patients with TBI or brain hemorrhage remains unclear. This study indicated that restarting aspirin had lower all-cause mortality in those patients 1 month after TBI episodes. In a nationwide cohort study of three Danish nationwide registries between 1997 and 2013, restarting anticoagulant treatment had lower all-cause mortality (HR, 0.55; 95% CI 0.37–0.82) 6 weeks after intracranial hemorrhage in patients with atrial fibrillation^24^. Moreover, Gonzalez-Perez et al.^25^ showed that significantly improved survival was associated with the current uses of starting low-dosage aspirin 30 days after a hemorrhagic stroke episode during a median of 6.4 years. On the contrary, aspirin discontinuation was associated with increased mortality (aHR 1.54; 95% CI 1.21–1.97)^25^. For those survivors of cerebral hemorrhage, recovery is slow. Minor patients could recover near-complete functioning within 30 days after the stroke; however, most recovery from intracranial hemorrhage occurs in the first few months after the stroke^26^. Therefore, the optimal timing for restarting antiplatelet agents 4 weeks after an episode of TBI or brain hemorrhage to decrease vascular risks is relatively safe, particularly in patients on antiplatelet therapy before the episode.

Our study’s strengths are the large sample size, the analysis of a national database, the consideration of the cumulative dose effect of drug use, and the subgroup analysis. This study minimized the selection bias and confounding factors by all matching the control group for age, sex, comorbidities, and medications. Despite its strengths and novelty, this study has some limitations that require clarifications. First, NHIRD does not provide laboratory data for further investigation. Second, lifestyle and personal habits cannot be obtained from the NHIRD. Third, previous stroke etiology, TBI severity, and skull fracture may have a bias in the use of aspirin. Forth, given that this study was an observational study of drug epidemiology and not a randomized controlled trial, there may have been allocation bias and prescription bias. Although a randomized controlled trial is the best way to demonstrate pharmaceuticals’ effects, most medical research is still conducted using drug epidemiology^27–29^. There are many situations where a randomized controlled trial is unsuitable. For example, there may be unethical occurrences, like adverse effects or other outcomes, studying drug interactions, a genetic disposition to diseases, and examining drug overdose.

## Conclusions

This study revealed that restarting aspirin use is associated with reducing the occurrence of secondary stroke (ischemic and hemorrhagic) and all-cause mortality 4 weeks after a TBI episode (including intracranial hemorrhage epsiodes) in patients with chronic stroke. Prospective studies are required before providing any definitive recommendations regarding the resumption's optimal timing and method.

## Supplementary Information

Supplementary Information.

## Data Availability

The datasets collected and analyzed in our study are available from the corresponding author (Wu-Chien Chien, email: chienwu@ndmctsgh.edu.tw) on reasonable request.
