# Supplementary material for: Risk of secondary stroke subsequent to restarting aspirin in chronic stroke patients suffering from traumatic brain injury in Taiwan
Source: Sci Rep. 2023 May 17;13:8001. doi: 10.1038/s41598-023-34986-z (PMC10192211; doi:10.1038/s41598-023-34986-z)
Supplement: Supplementary file 1 — Supplementary Information. [file 41598_2023_34986_MOESM1_ESM.doc]

**Supplemental Table S1.** Disease diagnosis codes according to the international classification of diseases, ninth revision, clinical modification (ICD-9-CM) and prescribed medications classified based on anatomical therapeutic chemical (ATC) classification.

| **Comorbidity / Medication** | **ICD-9-CM / ATC codes** |
| --- | --- |
| **Subject** |  |
| Aspirin | B01AC06 |
| Old stroke | 430, 432–437 |
| TBI | 800.1–800.4, 800.6–800.9, 801.1–801.4, 801.6–801.9, 803.1–803.4, 803.6–803.9, 804.1–840.4, 804.6–804.9, 851–852, 853.1. 854 |
| **Outcomes** |  |
| Ischemic stroke | 433, 434, 436 |
| Hemorrhagic stroke | 430, 432 |
| Myocardial infarction | 410–410.9, 412 |
| Arrhythmia | 427 |
| Out-of-hospital cardiac arrest | 427.5 |
| Infectious disease | 001–139, 790.7 |
| **Comorbodities** |  |
| Diabetes mellitus | 250–250.3, 250.7, 250.4–250.6 |
| CKD | 585.X |
| Stage 3 | 585.3 |
| Stage 4 | 585.4 |
| Stage 5 | 585.5 |
| Myocardial infarction | 410–410.9, 412 |
| Atrial fibrillation | 427.31 |
| Hypertension | 401–405 |
| Hyperlipidemia | 272 |
| Heart failure | 428–428.9 |
| Peripheral vascular disease | 443.9, 441–441.9, 785.4, V43.4 |
| Chronic pulmonary disease | 490–496, 505–505, 506.4 |
| Chronic liver disease | 571.2, 571.4–571.49, 571.5, 571.6, 456–456.21, 572.2–572.8 |
| Dementia | 290–290.9 |
| **Medication** |  |
| Clopidogrel | B01AC04 |
| Dipyridamole | B01AC07 |
| ACEI / ARB | C09 |
| Beta-2 blocker | C07 |
| CCB | C08 |
| Antiplatelet drug | B01AC |
| Statin | C10AA |
| NSAID | M01AA, M01AB, M01AC, M01AE, M01AG, M01AH, M01AX |
| Metformin | A10BA |
| Thiazolidinedione | A10BG |
| Sulfonylureas | A10BB |
| Alpha-glucosidase inhibitor | A10BF |
| DPP4is | A10BH, A10BD07, A10BD08, A10BD10, A10BD11 |
| Insulin | A10A |

Abbreviations: ACEI, angiotensin-converting enzyme inhibitor; ARB, angiotensin receptor blocker; CCB, calcium channel blockers; DDP4is, dipeptidyl peptidase 4 inhibitors; NSAID, nonsteroidal anti-inflammatory drug, TBI, traumatic brain injury.

**Supplemental Table S2.** Baseline Characteristics of Study Subjects before using the propensity score matching

| **Aspirin** | **With** (n = 15,035) |  | **Without** (n =103,266) | |  | ***P*** |
| --- | --- | --- | --- | --- | --- | --- |
| **Characteristics** | **mean ± SD** |  | **mean ± SD** | |  |  |
| **Age (years)** | 53.25 ± 19.74 |  | 58.72 ± 18.10 | |  | <0.001 |
|  | **No. (%)**  8,364 (55.63)  6,671 (44.37)  3,784 (25.17)  737 (4.90)  3,509 (23.34)  1,040 (6.92)  4,838 (32.18)  1,146 (7.62)  198 (1.32)  899 (5.98)  1,615 (10.74)  2,490 (16.56)  625 (4.16)  2,225 (14.80)  2,458 (16.35)  4,370 (29.07)  3,755 (24.98)  2,646 (17.60)  4,552 (30.28)  4,371 (29.07)  3,024 (17.60)  4,511 (30.00)  4,106 (16.24)  3,628 (24.13)  2,441 (16.24)  3,255 (21.65)  2,357 (15.68) |  | **No. (%)** | |  |  |
| **Sex** |  |  |  |  |  | 0.440 |
| Male |  |  | 57,591 (56.12)  45,315 (43.88)  37,123 (35.95)  5,971 (5.78)  14,790 (14.32)  5,555 (5.38)  29,704 (28.76)  8,244 (7.98)  1,107 (1.07)  5,018 (4.86)  7,980 (7.73)  10,136 (9.82)  3,022 (2.93)  24,801 (24.02)  11,372 (11.01)  34,989 (33.88)  27,080 (26.22)  24,883 (24.10)  21,077 (24.01)  33,705 (32.64)  19,976 (19.34)  22,838 (22.12)  28,452 (27.55)  20,174 (19.54)  15,207 (14.73)  29,035 (28.12)  12,043 (11.66) | |  |  |
| Female |  |  |  |  |  |  |
| **Comorbodities** |  |  |  |  |  |  |
| Diabetes mellitus |  |  |  |  |  | <0.001 |
| CKD |  |  |  |  |  | <0.001 |
| Myocardial infarction |  |  |  |  |  | <0.001 |
| Atrial fibrillation |  |  |  |  |  | <0.001 |
| Hypertension |  |  |  |  |  | <0.001 |
| Hyperlipidemia |  |  |  |  |  | <0.001 |
| Heart failure |  |  |  |  |  | <0.001 |
| Peripheral vascular disease |  |  |  |  |  | <0.001 |
| Chronic pulmonary disease |  |  |  |  |  | <0.001 |
| Chronic liver disease |  |  |  |  |  | <0.001 |
| Dementia |  |  |  |  |  | <0.001 |
| **Medication** |  |  |  |  |  |  |
| Clopidogrel |  |  |  |  |  | <0.001 |
| Dipyridamole |  |  |  |  |  | <0.001 |
| ACEI / ARB |  |  |  |  |  | <0.001 |
| Beta-2 blocker |  |  |  |  |  | <0.001 |
| CCB |  |  |  |  |  | <0.001 |
| Antiplatelet drug |  |  |  |  |  | <0.001 |
| Statin |  |  |  |  |  | <0.001 |
| NSAID |  |  |  |  |  | <0.001 |
| Metformin |  |  |  |  |  | <0.001 |
| Thiazolidinedione |  |  |  |  |  | <0.001 |
| Sulfonylureas |  |  |  |  |  | <0.001 |
| Alpha-glucosidase inhibitor |  |  |  |  |  | <0.001 |
| DPP4is |  |  |  |  |  | <0.001 |
| Insulin |  |  |  |  |  | <0.001 |

**Supplemental Table S3.** Standardized differences before and after using the propensity score matching.

| **Matching, (With aspirin)** | **Before** (n =103,266) |  | **After** (n = 60,140) | |  | **SMD** |
| --- | --- | --- | --- | --- | --- | --- |
| **Characteristics** | **mean ± SD** |  | **mean ± SD** | |  |  |
| **Age (years)** | 58.72 ± 18.10 |  | 53.12 ± 19.22 | |  | 0.025 |
|  | **No. (%)**  57,591 (56.12)  45,315 (43.88)  37,123 (35.95)  5,971 (5.78)  14,790 (14.32)  5,555 (5.38)  29,704 (28.76)  8,244 (7.98)  1,107 (1.07)  5,018 (4.86)  7,980 (7.73)  10,136 (9.82)  3,022 (2.93)  24,801 (24.02)  11,372 (11.01)  34,989 (33.88)  27,080 (26.22)  24,883 (24.10)  21,077 (24.01)  33,705 (32.64)  19,976 (19.34)  22,838 (22.12)  28,452 (27.55)  20,174 (19.54)  15,207 (14.73)  29,035 (28.12)  12,043 (11.66) |  | **No. (%)** | |  |  |
| **Sex** |  |  |  |  |  | 0.009 |
| Male |  |  | 33,486 (55.63)  26,684 (44.37)  15,382 (25.58)  2,859 (4.75)  13,977 (23.24)  4,192 (6.97)  19,320 (32.13)  4,528 (7.53)  881 (1.46)  3,614 (6.01)  6,429 (10.69)  9,683 (16.10)  2,406 (4.00)  8,774 (14.59)  9,520 (15.83)  17,542 (29.17)  14,771 (24.56)  14,574 (24.23)  18,312 (30.45)  17,662 (29.37)  10,765 (17.90)  18,142 (30.17)  17,169 (28.55)  14,643 (24.35)  9,758 (16.23)  12,973 (21.57)  9,363 (15.57) | |  |  |
| Female |  |  |  |  |  |  |
| **Comorbodities** |  |  |  |  |  |  |
| Diabetes mellitus |  |  |  |  |  | 0.186 |
| CKD |  |  |  |  |  | 0.073 |
| Myocardial infarction |  |  |  |  |  | 0.262 |
| Atrial fibrillation |  |  |  |  |  | 0.137 |
| Hypertension |  |  |  |  |  | 0.124 |
| Hyperlipidemia |  |  |  |  |  | 0.025 |
| Heart failure |  |  |  |  |  | 0.036 |
| Peripheral vascular disease |  |  |  |  |  | 0.103 |
| Chronic pulmonary disease |  |  |  |  |  | 0.296 |
| Chronic liver disease |  |  |  |  |  | 0.307 |
| Dementia |  |  |  |  |  | 0.104 |
| **Medication** |  |  |  |  |  |  |
| Clopidogrel |  |  |  |  |  | 0.128 |
| Dipyridamole |  |  |  |  |  | 0.065 |
| ACEI / ARB |  |  |  |  |  | 0.176 |
| Beta-2 blocker |  |  |  |  |  | 0.109 |
| CCB |  |  |  |  |  | 0.033 |
| Antiplatelet drug |  |  |  |  |  | 0.207 |
| Statin |  |  |  |  |  | 0.146 |
| NSAID |  |  |  |  |  | 0.030 |
| Metformin |  |  |  |  |  | 0.082 |
| Thiazolidinedione |  |  |  |  |  | 0.019 |
| Sulfonylureas |  |  |  |  |  | 0.063 |
| Alpha-glucosidase inhibitor |  |  |  |  |  | 0.049 |
| DPP4is |  |  |  |  |  | 0.074 |
| Insulin |  |  |  |  |  | 0.038 |

SMD = Standardized mean difference

**Supplemental Table S4.** Subgroup analysis of the association between aspirin and the risk of hospitalization of ischemic stroke and hemorrhagic stroke.

|  | | **With aspirin** (n = 15,035) | | |  | **Without aspirin** (n = 60,140) | | |  | **With aspirin *vs.* without aspirin** *(Reference)* | | | |
| --- | --- | --- | --- | --- | --- | --- | --- | --- | --- | --- | --- | --- | --- |
| **Subgroups analysis** | | **Events** | **PYs** | **Rate** |  | **Events** | **PYs** | **Rate** |  | **aHR** | **95% CI** | **95% CI** | ***P*** |
| **Overall** | | 1,340 | 139,744.92 | 9.59 |  | 6,046 | 555,407.13 | 10.89 |  | 0.671 | 0.591 | 0.731 | <0.001 |
| **Diabetes mellitus** | Without | 668 | 103,643.66 | 6.45 |  | 3,094 | 412,100.01 | 7.51 |  | 0.614 | 0.544 | 0.673 | <0.001 |
|  | With | 672 | 36,101.26 | 18.61 |  | 2,952 | 143,307.12 | 20.60 |  | 0.691 | 0.604 | 0.755 | <0.001 |
| **CKD** | Without | 985 | 132,905.68 | 7.41 |  | 4,530 | 527,852.95 | 8.58 |  | 0.610 | 0.537 | 0.661 | <0.001 |
|  | With | 355 | 6,839.24 | 51.91 |  | 1,516 | 27,554.18 | 55.02 |  | 0.854 | 0.751 | 0.928 | <0.001 |
| **Myocardial infarction** | Without | 691 | 105,674.03 | 6.54 |  | 3,312 | 420,405.85 | 7.88 |  | 0.646 | 0.569 | 0.701 | <0.001 |
|  | With | 649 | 34,070.89 | 19.05 |  | 2,734 | 135,001.28 | 20.25 |  | 0.700 | 0.616 | 0.763 | <0.001 |
| **Atrial fibrillation** | Without | 1,159 | 129,011.68 | 8.98 |  | 5,326 | 514,581.67 | 10.35 |  | 0.642 | 0.566 | 0.697 | <0.001 |
|  | With | 181 | 10,733.24 | 16.86 |  | 720 | 40,825.46 | 17.64 |  | 0.856 | 0.752 | 0.950 | 0.001 |
| **Clopidogrel user** | Without | 825 | 118,771.81 | 6.95 |  | 3,705 | 472,365.88 | 7.84 |  | 0.680 | 0.600 | 0.742 | <0.001 |
|  | With | 515 | 20,973.11 | 24.56 |  | 2,341 | 83,041.25 | 28.19 |  | 0.652 | 0.569 | 0.708 | <0.001 |
| **Dipyridamole user** | Without | 706 | 116,909.64 | 6.04 |  | 3,315 | 464,766.91 | 7.13 |  | 0.654 | 0.576 | 0.705 | <0.001 |
|  | With | 634 | 22,835.28 | 27.76 |  | 2,731 | 90,640.22 | 30.13 |  | 0.688 | 0.604 | 0.745 | <0.001 |

PYs = Person-years; Rate: per 1,000 PYs; aHR = Adjusted Hazard ratio: Adjusted for gender, age, Comorbodities, and medications; CI = confidence interval

**Supplemental Table S5. Subgroup analysis of the association between aspirin and risk of all-cause mortality.**

|  | | **With aspirin** (n = 15,035) | | |  | **Without aspirin** (n = 60,140) | | |  | **With aspirin *vs.* without aspirin** *(Reference)* | | | |
| --- | --- | --- | --- | --- | --- | --- | --- | --- | --- | --- | --- | --- | --- |
| **Subgroups analysis** | | **Events** | **PYs** | **Rate** |  | **Events** | **PYs** | **Rate** |  | **aHR** | **95% CI** | **95% CI** | ***P*** |
| **Overall** | | 1,350 | 147,812.35 | 9.13 |  | 5,956 | 592,206.11 | 10.06 |  | 0.840 | 0.720 | 0.946 | <0.001 |
| **Diabetes mellitus** | Without | 895 | 109,360.06 | 8.18 |  | 4,008 | 438,835.89 | 9.13 |  | 0.812 | 0.704 | 0.928 | <0.001 |
|  | With | 455 | 38,452.29 | 11.83 |  | 1,948 | 153,370.22 | 12.70 |  | 0.876 | 0.742 | 0.988 | 0.039 |
| **CKD** | Without | 995 | 140,543.87 | 7.08 |  | 4,483 | 562,822.54 | 7.97 |  | 0.819 | 0.696 | 0.926 | <0.001 |
|  | With | 355 | 7,268.48 | 48.84 |  | 1,473 | 29,383.57 | 50.13 |  | 0.913 | 0.775 | 1.029 | 0.105 |
| **Myocardial infarction** | Without | 1,210 | 111,513.10 | 10.85 |  | 5,338 | 448,457.21 | 11.90 |  | 0.834 | 0.710 | 0.940 | <0.001 |
|  | With | 140 | 36,299.25 | 3.86 |  | 618 | 143,748.90 | 4.30 |  | 0.880 | 0.745 | 0.993 | 0.047 |
| **Atrial fibrillation** | Without | 1,024 | 136,953.08 | 7.48 |  | 4,642 | 549,195.23 | 8.45 |  | 0.820 | 0.698 | 0.927 | <0.001 |
|  | With | 326 | 10,859.27 | 30.02 |  | 1,314 | 43,010.88 | 30.55 |  | 0.893 | 0.760 | 1.011 | 0.058 |
| **Clopidogrel user** | Without | 1,053 | 125,463.39 | 8.39 |  | 4,464 | 504,099.86 | 8.86 |  | 0.885 | 0.755 | 0.961 | 0.010 |
|  | With | 297 | 22,348.96 | 13.29 |  | 1,492 | 88,106.25 | 16.93 |  | 0.697 | 0.597 | 0.785 | <0.001 |
| **Dipyridamole user** | Without | 979 | 123,547.38 | 7.92 |  | 4,465 | 495,062.86 | 9.02 |  | 0.816 | 0.690 | 0.926 | <0.001 |
|  | With | 371 | 24,264.97 | 15.29 |  | 1,491 | 97,143.25 | 15.35 |  | 0.904 | 0.772 | 0.956 | 0.004 |

PYs = Person-years; Rate: per 1,000 PYs; aHR = Adjusted Hazard ratio: Adjusted for gender, age, Comorbodities, and medications; CI = confidence interval

**Supplement Table S6.** Causes of all-cause mortality assessed by Cox regression.

|  | **With aspirin *vs.* without aspirin** *(Reference)* | | | |
| --- | --- | --- | --- | --- |
| **Causes of mortality** | **aHR** | **95% CI** | **95% CI** | ***P*** |
| All-caused mortality | 0.840 | 0.720 | 0.946 | <0.001 |
| Ischemic stroke | 0.745 | 0.618 | 0.831 | <0.001 |
| Hemorrhage stroke | 0.702 | 0.610 | 0.771 | <0.001 |
| Myocardial infarction | 0.764 | 0.689 | 0.860 | <0.001 |
| Arrhythmia | 0.937 | 0.778 | 1.042 | 0.083 |
| Out-of-hospital cardiac arrest | 1.093 | 0.965 | 2.122 | 0.596 |
| Infectious disease | 0.969 | 0.934 | 1.434 | 0.225 |

aHR = Adjusted Hazard ratio: Adjusted for gender, age, Comorbodities, and medications; CI = confidence interval

**Supplemental Table S7.** Comparing hospitalization of stroke and mortality between chronic stroke patients with severe traumatic brain injury (with long-stay hospitalization or surgical treatment) with and without restarting aspirin use in the Cox model with competing risks.

|  |  | **With aspirin *vs.* without aspirin** *(Reference)* | | | | | | | | |
| --- | --- | --- | --- | --- | --- | --- | --- | --- | --- | --- |
|  | **Fine and Gray competing risk** | **No competing in the model** | | | |  | **Competing risk in the model** | | | |
| **TBI** | **Outcomes** | **aHR** | **95% CI** | **95% CI** | ***P*** |  | **aSHR** | **95% CI** | **95% CI** | ***P*** |
|  | Hospitalization of stroke | 0.671 | 0.591 | 0.731 | <0.001 |  | 0.679 | 0.606 | 0.742 | <0.001 |
|  | Hospitalization of ischemic stroke | 0.679 | 0.612 | 0.745 | <0.001 |  | 0.694 | 0.621 | 0.756 | <0.001 |
|  | Hospitalization of hemorrhage stroke | 0.631 | 0.538 | 0.720 | <0.001 |  | 0.642 | 0.549 | 0.723 | <0.001 |
|  | All-caused mortality | 0.840 | 0.720 | 0.946 | <0.001 |  | - | - | - | - |
| **Severe TBI** | **Outcomes** | aHR | 95% CI | 95% CI | P |  | aSHR | 95% CI | 95% CI | P |
|  | Hospitalization of stroke | 0.621 | 0.559 | 0.691 | <0.001 |  | 0.630 | 0.572 | 0.711 | <0.001 |
|  | Hospitalization of ischemic stroke | 0.643 | 0.508 | 0.703 | <0.001 |  | 0.653 | 0.593 | 0.729 | <0.001 |
|  | Hospitalization of hemorrhage stroke | 0.599 | 0.493 | 0.676 | <0.001 |  | 0.617 | 0.501 | 0.688 | <0.001 |
|  | All-caused mortality | 1.064 | 0.835 | 1.488 | 0.767 |  | - | - | - | - |

aHR = Adjusted Hazard ratio: Adjusted for gender, age, Comorbodities, and medications; CI = confidence interval; aSHR = Adjusted Subdistribution Hazard Ratio: Adjusted for gender, age, Comorbodities, and medications
